# Protection acquired upon intraperitoneal group a *Streptococcus* immunization is independent of concurrent adaptive immune responses but relies on macrophages and IFN-γ

**DOI:** 10.1080/21505594.2025.2457957

**Published:** 2025-02-08

**Authors:** Shiva Emami, Elsa Westerlund, Thiago Rojas Converso, Bengt Johansson-Lindbom, Jenny J Persson

**Affiliations:** Department of Experimental Medical Science, Lund University, Lund, Sweden

**Keywords:** Group A Streptococcus, immunization, protective immunity, adaptive immune memory, innate immune memory

## Abstract

Group A *Streptococcus* (GAS; *Streptococcus pyogenes*) is an important bacterial pathogen causing over 700 million superficial infections and around 500.000 deaths due to invasive disease or severe post-infection sequelae yearly. In spite of this major impact on society, there is currently no vaccine available against this bacterium. GAS strains can be separated into >250 distinct *emm* (M)-types, and protective immunity against GAS is believed to in part be dependent on type-specific antibodies. Here, we analyse the nature of protective immunity generated against GAS in a model of intraperitoneal immunization in mice. We demonstrate that multiple immunizations are required for the ability to survive a subsequent lethal challenge, and although significant levels of GAS-specific antibodies are produced, these are redundant for protection. Instead, our data show that the immunization-dependent protection in this model is induced in the absence of B and T cells and is accompanied by the induction of an altered acute cytokine profile upon subsequent infection, noticeable e.g. by the absence of classical pro-inflammatory cytokines and increased IFN-γ production. Further, the ability of immunized mice to survive a lethal infection is dependent on macrophages and the macrophage-activating cytokine IFN-γ. To our knowledge these findings are the first to suggest that GAS may have the ability to induce forms of trained innate immunity. Taken together, the current study proposes a novel role for the innate immune system in response to GAS infections that potentially could be leveraged for future development of effective vaccines.

## Introduction

Group A *Streptococcus* (GAS, *Streptococcus pyogenes*) is one of our major circulating bacterial pathogens, causing >700 million uncomplicated infections and ~ 500.000 deaths annually [1]. The vast majority of GAS infections are superficial (e.g. tonsillitis or impetigo) and occur in children. In contrast, uncommon devastating infections like necrotizing fasciitis or toxic shock syndrome occur predominantly in the adult or elderly population [1]. In rare cases, recurring and untreated mild infections may give rise to the autoimmune syndrome acute rheumatic fever (ARF), which may progress to life-threatening rheumatic heart disease [2,3]. ARF is believed to at least in part be caused by immune responses generated against the GAS M protein that cross-react with e.g. cardiac myosin in the host [2]. The M protein is a major virulence factor and contains a hypervariable region (HVR) that allows for the differentiation of GAS strains into >250 *emm* types [4] and is also target for protective, type-specific antibodies [5]. The extensive strain variability, immuno-subdominance of the HVR [6,7] and the risk of vaccine components triggering ARF [8] are all major challenges in GAS vaccine development. There is currently no vaccine available against GAS, however several M-protein and non-M-protein-based vaccine candidates are investigated in early phase clinical trials [9,10].

Immunological memory is formed from highly specific B and/or T cells. During the resolution phase after an infection, expanded populations of specific B and T cells contract and leave behind memory cells with heightened activation potential. Recent advances however have shown that the spectrum of lasting infection- or vaccine-induced immune protection is broader than previously appreciated. This includes several forms of imprinted memory-like functions, often driven by metabolic and epigenetic changes in innate immune cell populations [11]. Out of these innate immune memory forms, so-called “trained immunity” (increased heterologous responsiveness) has been most extensively studied, predominantly in myeloid populations, but also in lymphoid NK cells [12], other ILCs [13], and γδ T cells [14] and non-immune cells like fibroblasts [15] and epithelial cells [16]. Of note, when trained immunity is induced in cells directly in peripheral tissues it is referred to as “peripheral trained immunity,” while adaptations in precursors in the bone marrow is known as “central trained immunity” [11]. In addition to trained immunity, the immune refractory phenomenon of innate cross-tolerance [17], and adaptive-like specific memory in NK cells [18] has been described.

Understanding the mechanisms underlying natural or experimentally induced immune protection against GAS has been a topic of interest for decades, and most attention has been devoted to the type-specific immunity GAS infections may confer [19–21]. In addition, it also seems that a more general protection may be present in adults as compared to children [22,23], which to some degree might be explained by partial cross-protection observed within clusters of similar GAS strains building up over time [24,25]. Whether or not innate immune memory forms may contribute to the broader protection against GAS infection observed with age has not been investigated, and neither has the potential ability of GAS itself to induce memory phenotypes in innate immune cells.

In this study, we investigate protective immunity conferred by intraperitoneal (ip) immunization of mice with intact heat killed (HK) GAS. We find that repeated injections are required to establish protection, and that protection induced through this immunization regimen seems to be independent of canonical adaptive immune responses. Our work instead indicates that GAS immunizations may generate a form of imprinted immunity in innate immune cell populations.

## Materials and methods

### Bacterial strains and culture conditions

The *S. pyogenes* strain 90–226 is an M1 serotype strain originally isolated from a sepsis patient [26] and is referred to as GAS-M1 in this study. Bacterial cultures were grown over night without shaking in Todd-Hewitt broth supplemented with 0.2% yeast extract (THY) in 5% CO_2_ at 37°C, and re-inoculated and grown until they reached optical density (OD) of 0.8–1.

### Mice

Female C57Bl/6 (B6) mice were purchased from Taconic and IFN-*λ*-KO mice (B6.129S7-Ifng^tm1Ts^/J) were originally from The Jackson Laboratory. The CD4-Cre x Bcl6^fl/fl^ strain was generated by crossing CD4-Cre (B6.CgTg(Cd4-cre)1Cwi/BfluJ) and Bcl6^fl/fl^ (B6.129S(FVB)Bcl6 ^tml.1Dent^/J) mice. Animals with the CD4-Cre x Bcl6^fl/fl^ genotype do not develop T follicular helper (Tfh) cells, as they lack the Tfh master transcription factor Bcl6 in CD4^+^ T-cells. Rag1-KO mice on a B6 background were originally from The Jackson Laboratory. To ensure consistency and reliability in our results, we used the C57Bl/6J background when comparing genetically modified mice with the B6 strain. To reduce potential source of bias variability, we consistently selected experimental mice of the same gender and age.

For experiments using B6 mice only, 8-week-old females were used while genetically modified mice were both female and male, aged 8–12 weeks. All animals were bred (except B6) and maintained at the animal facility at the Lund University Biomedical Center. Experiments were performed in accordance with protocols approved by the Lund/Malmö Animal Ethics Committee, permit numbers: 7342/2017 and 07178/2020 and follow the ARRIVE guidelines for animal research.

### Immunizations and infections

To generate heat killed (HK) or formalin fixed (FF) bacteria, log phase cultures were washed with PBS and incubated at 60°C for two hours or fixed with 1% formalin to achieve complete killing. Killing was confirmed by blood agar plating. Suspensions of killed bacteria were diluted to the appropriate concentration and stored at −20°C (HK) or 4°C (FF). Mice were injected with 200 µl suspensions containing 10^8^ HK or FF GAS-M1 once, or three times ip at three-week intervals and bled 19 days after each injection. Control mice were injected with PBS only.

For the protection experiments, a lethal dose of 10^8^ CFU live GAS-M1 was administered intraperitoneally (ip) three weeks after the final immunization injection. Due to the unavoidable variability in the *de facto* effective dose for each experimental occasion, survival levels in immunized and control mice both exhibit some variability between experiments. To prevent any bias in assessing moribundity, the cages were blinded following injection. Mice exhibiting shaking, piloerection, and immobility despite provocation were deemed moribund and euthanized. Monitoring was conducted every four hours for the first two days post-challenge, and thereafter twice per day.

### Serum antibody titer measurements

To assess antibody titres in mouse serum, HiBond ELISA plates were coated with 10^7^ CFU of GAS-M1 bacteria killed in PBS containing 0.05% Sodium azide. These coated plates were incubated overnight at 4°C and on the following day incubated with serially diluted serum samples obtained from both immunized and non-immunized mice, or PBS only for control of background readings. Background control wells consistently gave a signal similar to or below the serum samples from the mice injected with PBS only (data not shown). Captured antibodies were detected using biotinylated goat anti-mouse IgG (Southern Biotech, CAT: 1036–08), or IgM (BioLegend, RMM-1) and streptavidin-HRP (BioLegend, Cat 405,210). HRP was activated using TMB substrate (Sigma, T8768). All samples were run in duplicate and presented values represent the average of duplicates. BSA-coated wells served as negative controls.

### Detection of bacterial counts, immune cells, and cytokines in different organs of infected mice

On the selected time points (4 or 24 hours) post infection, animals were sacrificed, and 5 ml sterile PBS was injected into the peritoneal cavity. The abdominal area was massaged briefly and the ip wash was extracted using a syringe and kept on ice. The total number of recovered white blood cells was determined in a Sysmex haematology analyser (Sysmex), and bacterial numbers were determined by blood agar plate culture and manual counting. Blood was collected from the heart and mixed with 0.5 M EDTA to prevent coagulation. The spleen was removed, weighed, and homogenized using a 70 μm cell strainer. Serial dilutions of blood and spleen samples were cultured on blood agar plates for determination of bacterial loads. Immune cells were identified in ip wash, blood or homogenized spleen by flow cytometry, using the following reagents and fluorochrome conjugated antibodies: Live/dead fixable APC-Cy7 (Invitrogen, L34975), Anti-CD3-AF700 (BD, 17A2), Anti-NK-1.1-AF700 (BioLegend, PK136), Anti-CD19-AF700 (BioLegend, 6D5), Anti-CD11b-BV605 (BioLegend, M1/70), Anti-CD11c-PE-CY7 (Invitrogen, N418), Anti-MHC-II-BV421 (BioLegend, M5/114.15.2), Anti-Gr-1-BV510 (BioLegend, RB6-8C5), Anti-Ly6C-PerCP-Cy5.5 (BioLegend, HK1.4), Anti- CD64-AF647 (BD Pharmigen, X54–5/7.1), Anti-Siglec-F-PE (BD Pharmigen, E50–2440). Cytokine concentrations were measured using CBA (single-colour antibodies; BD Biosciences).

### Depletion of T cells and macrophages

For T cell-depletion experiments, mice were injected ip with a depleting anti-CD4 antibody (InVivoMab, GK1.5) two days before infection, and on post infection days + 1 and + 4. Control mice received isotype control antibody (InVivoMab, LTF-2) only. Spleen cells were recovered as described above and T cells were analysed by BD LSR II Flow Cytometer using the following reagents and fluorochrome conjugated antibodies: Live/dead fixable APC-Cy7 (Invitrogen, L34975), Anti-CD3-BV605 (BD, 17A2), Anti-CD4-FITC (eBioscience, RM4–5), Anti-CD8-BV650 (BD, 53–6.7).

To deplete macrophages, immunized mice were injected ip with 500 μl clodronate liposomes (5 mg/ml; LIPOSOMA BV, NL) three days prior to and one day post infection. Immunized control mice received PBS liposomes at the same time points. To identify cells in the peritoneum (ip wash, as described above), the following reagents/antibodies were used: Anti-CD3-AF700 (BioLegend, 17A2), Anti-CD11b-BV786 (BD Bioscience, M1/70), Anti-CD19-BV711 (BioLegend, 6D5), Anti-CD64-APC (BD Bioscience, X54–5/7.1), Anti-F4/80-BV421 (Biolegend, BM8), Anti-Ly6C-FITC (BioLegend, HK1.4), Anti-Ly6G-PE (BioLegend,1A8), and live/dead PI-PE-CF594 (Invitrogen, CAT P3566).

### Statistical analysis

Data were analysed using Prism version 10.0 (GraphPad Software). Analysis of statistical significance was performed using one-way ANOVA with Tukey’s multiple comparisons test for three or more groups, or t-test for two unpaired groups. Comparison of survival curves was done by Kaplan-Meier survival test with Log-rank (Mantel-Cox), comparing the median survival hours for each group. Differences were considered significant when *p* ≤ 0.05 (*p ≤ 0.05, **p < 0.01, ***p < 0.001 and ****p < 0.0001).

## Results

### Repeated intraperitoneal immunization with heat-killed GAS-M1 confers protection

**Figure 1.**
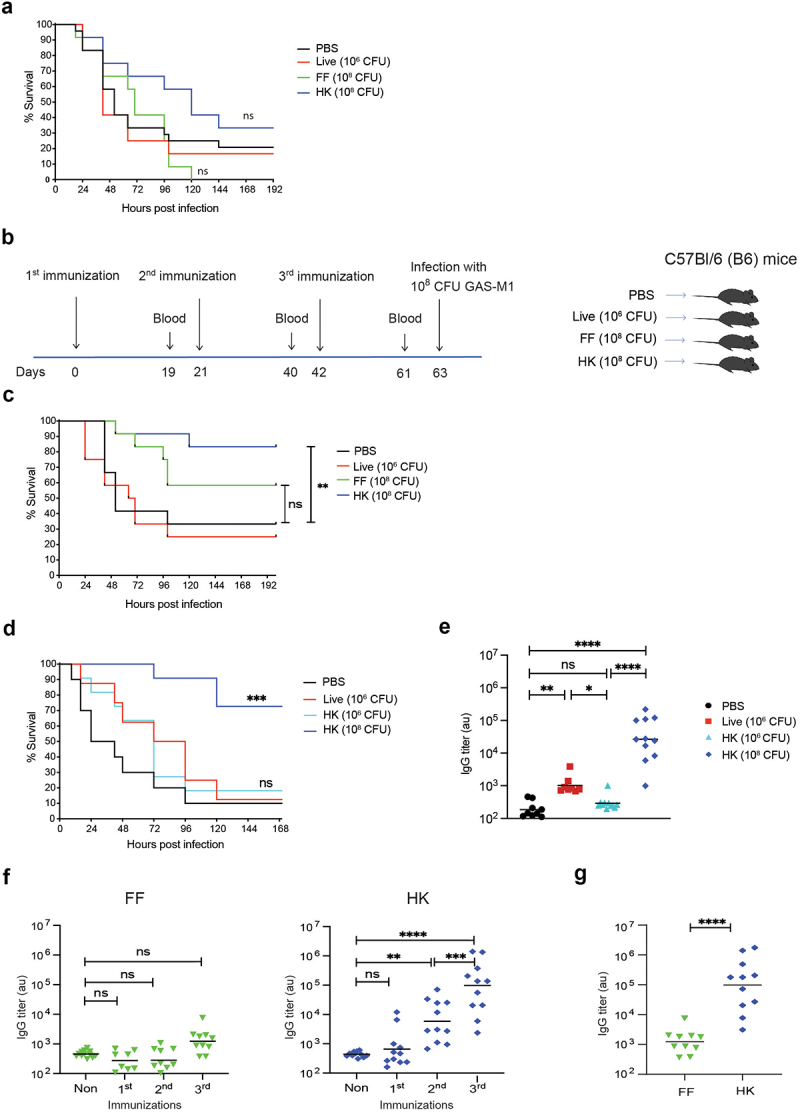
Protective immunity after repeated intraperitoneal immunization with HK GAS-M1. (a-c) B6 mice (*n* = 12 per group) were immunized ip with one or three doses of HK GAS-M1 (10^8^ CFU), FF GAS-M1 (10^8^ CFU), or live GAS-M1 (10^6^ CFU) with three weeks interval between each dose. Three weeks after completed immunization schedule, mice were challenged ip with a lethal dose of GAS-M1 (10^8^ CFU) and monitored for 8 days. During the immunization phase, the researcher was aware of the group assignments. However, during and after the infection, the cages were blinded, ensuring that the survival or death of the mice was not linked to their groups until the end of the experiment. (a) Kaplan-Meier survival plot with log-rank tests following immunization with one dose only. (b) Experimental layout for experiments based on three consecutive immunizations. (c) Kaplan-Meier survival plot with log-rank tests for mice immunized with three consecutive doses. (d–e). B6 mice (*n* = 10–11 per group) were immunized as above with live (10^6^ CFU; highest non-pathological dose possible), or HK (10^6^ and 10^8^ CFU, respectively) bacteria, and then challenged ip with a lethal dose and monitored as above. (d) Kaplan-Meier survival plot with log-rank tests. (e) Serum level of IgG against intact GAS-M1 19 days after the third immunization (mice in 1D). (f) Serum level of IgG against intact GAS-M1 19 days after each dose of FF and HK GAS-M1 (mice in 1C). Each immunization type was performed at least twice in independent experiments, and presented data is from one representative experiment. Control B6 mice received PBS only. (g) Direct comparison of serum levels of IgG against intact GAS-M1 after third dose of FF and HK GAS-M1 (1F). Statistical significance was analysed by one-way ANOVA with Tukey’s multiple comparisons test. *p < 0.05, **p < 0.01, ***p < 0.001 and ****p < 0.0001. ns = not significant, au = arbitrary units.

The route of infection or vaccination strongly affects the quality and quantity of immune responses, both in humans and in experimental murine models [27,28], including models of GAS infection [29–31]. Although not used in human vaccination practice, intraperitoneal (ip) injections are commonly used in murine research models. Both to model systemic infection, as they support rapid dissemination of the pathogen, and for analysis of immune responses during immunization/vaccination regimens as they accommodate self-drain of large antigens (e.g. bacteria) to the lymphatic system, and subsequently to the blood [32]. To identify an immunization strategy that would provide protective immunity against a systemic (ip) GAS challenge, we first conducted a set of one-dose immunizations as follows: 1. 10^6^ CFU live GAS-M1 (highest possible non-lethal dose), 2. 10^8^ CFU formalin-fixed (FF) GAS-M1, and 3. 10^8^ CFU HK GAS-M1, were injected ip into female C57Bl/6 (B6) mice. Control mice received PBS only. Three weeks after immunization, mice were challenged ip with a lethal dose of 10^8^ CFU GAS-M1 and monitored for moribundity. As neither of these immunizations provided protection above control levels (Figure 1a), we then designed an extended protocol employing the same immunization formulations (10^6^ live, or 10^8^ FF or HK GAS-M1) but using three injections with three-week intervals. In addition, all animals were bled 19 days after each immunization (Figure 1b). Three injections with live bacteria did still not provide increased protection as compared to control mice receiving PBS only during the immunization scheme. Mice immunized three times with FF bacteria displayed a non-significant trend towards increased survival as compared to control mice, while immunizations using HK GAS-M1 provided significant protection (Figure 1c).

We were intrigued by the apparent inability of live bacteria to induce protection and measurable antibody responses but were unable to make proper comparisons to the FF or HK bacterial injections because of the differences in distributed dose (FF=HK: 10^8^, live: 10^6^). Therefore, we immunized mice with the same lower dose of HK and live GAS-M1 and observed that at this lower dose, HK GAS-M1 similarly failed to induce both protection and specific IgG after three immunizations (Figure 1d,e). These findings show that the provided protection is dose dependent, and thus prevent us from drawing conclusions regarding the intrinsic ability or inability of live bacteria to confer protective immunity using this regimen.

As antibodies commonly are important for protection against extracellular bacterial pathogens, we followed the serum GAS-M1-specific IgG levels through the immunization regimens. While FF bacteria failed to stimulate an increase in GAS-M1-specific IgG after three immunizations, HK bacteria induced significantly elevated levels already after two immunizations (Figure 1f). After the third HK dose, levels increased further resulting in ~2 log higher levels of specific IgG than that observed for immunizations using FF bacteria (Figure 1g). In agreement with the lack of protection observed after the one-dose immunization regimens, neither of the formulations promoted IgG generation after a single dose. Based on these findings, we opted to use a dose of 10^8^ CFU of HK GAS-M1 for all subsequent immunization experiments.

### Protective immunity after intraperitoneal immunization does not rely on germinal center-derived antibodies

**Figure 2.**
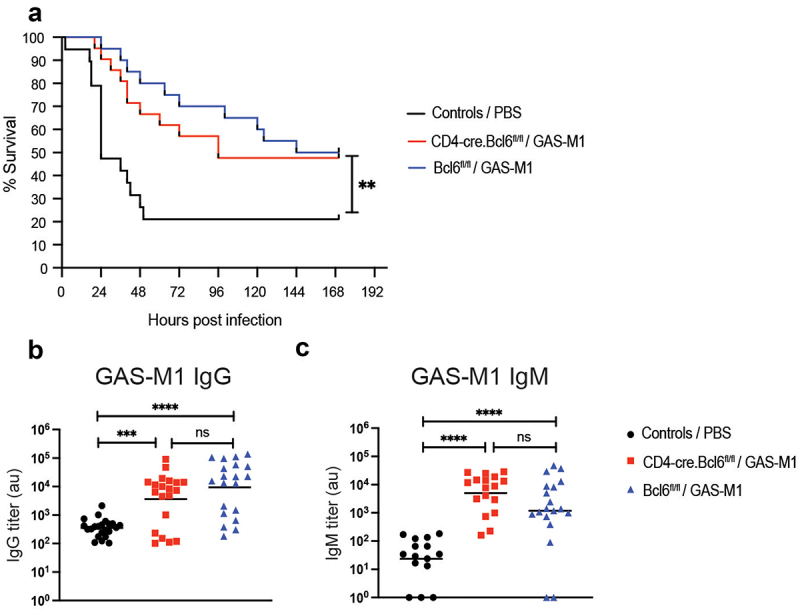
Protection conferred through the intraperitoneal immunization regimen does not depend on gc-derived antibodies. CD4-Cre.Bcl6^fl/fl^ (*n* = 20) and Bcl6^fl/fl^ (*n* = 21) mice received three ip immunization injections with HK GAS-M1. A control group (*n* = 19), including mice of each genotype, received three doses of PBS only. Three weeks after the last dose, mice were challenged ip with 10^8^ CFU of live GAS-M1 and the animals were monitored for seven days post-infection. Pooled results from three separate experiments are shown. (a) Kaplan-Meier survival plot with log-rank tests. (b-c) Serum level of IgG (b) and IgM (c) against intact GAS-M1 19 days after the third immunization and two days prior to administration of the lethal dose. Statistical significance was analysed by one-way ANOVA with Tukey’s multiple comparisons test. **p < 0.01, ***p < 0.001 and ****p < 0.0001. ns = not significant, au = arbitrary units.

High affinity and isotype switched antibody responses develop mostly within germinal centres (GC), histological structures appearing in B cell follicles of secondary lymphoid organs after infections or vaccination. GC B cell responses are dependent on T follicular helper (Tfh) cells, a subset of CD4^+^ T cells, distinct from “canonical” Th effector cells, that are functionally specialized in providing B cell help [33]. Although isotype switching can occur also during T cell-independent B cell activation, these responses are mostly associated with production of low-affinity IgM [34]. To investigate the role for GC-derived antibodies in the protection acquired through ip immunization with HK GAS-M1, we employed the CD4-Cre.Bcl6^fl/fl^ mouse model. In this model, Cre^+^ mice lack the transcription factor Bcl6 in T cells, preventing Tfh cell differentiation and GC formation [35–37], without affecting other Th effector mechanisms [38]. We immunized CD4-Cre.Bcl6^fl/fl^ and Bcl6^fl/fl^ (Cre^−^, *Bcl6* sufficient) mice with HK GAS-M1 and were surprised to observe that even though there seemed to be a trend towards decreased survival in immunized animals lacking *Bcl6* in T cells, this was not a statistically significant difference (Figure 2a). This result indicates that the generation of GC responses, which can only occur in the Bcl6^fl/fl^ (Cre^−^) mice, is redundant for protection. Similarly, regardless of the ability to form GCs, all animals exhibited comparable levels of IgG (Figure 2b) and IgM (Figure 2c) after immunization, although we observed trends indicating decreased IgG and increased (potentially compensatory) IgM levels. Based on our significant observations, we however conclude that GC formation and GC-derived antibodies do not seem to be essential for protection in this model, and that the generated antibodies may in fact predominantly be non-GC-derived.

### Protective immunity does not require T cell help during lethal GAS-M1 infection in immunized animals

**Figure 3.**
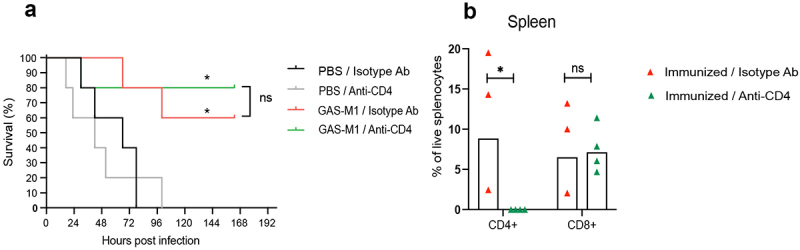
Depletion of CD4 T cells prior to lethal challenge does not affect survival of mice previously immunized with HK GAS-M1. B6 mice were immunized ip with three doses of HK GAS-M1 with three weeks interval between each dose (*n* = 10). Control B6 mice received 3 × PBS only (*n* = 10). Depleting anti-CD4 monoclonal antibody was administered to half of the mice in each group 2 days before as well as 1 and 4 days after administration of a lethal dose GAS-M1. Remaining mice in each group instead received an isotype control antibody by the same schedule as used for anti-CD4 treatment. Three weeks after the last immunization, all mice were infected ip with a lethal dose of GAS-M1 (10^8^ CFU) and then monitored for seven days. (a) Kaplan-Meier survival plot with log-rank tests with significant differences in relation to controls receiving PBS and isotype control antibody (Pbs/isotype Ab) indicated. (b) Frequencies of CD4^+^ and CD8^+^ T cells in the spleen of immunized mice receiving anti-CD4 or control monoclonal antibodies and surviving the lethal dose until the experimental endpoint seven days post infection. *p < 0.05, ns = not significant.

In addition to providing help to B cells, memory Th cell subsets can facilitate clearance of bacteria through cytokine-mediated effects on myeloid cells, including macrophages and neutrophils [39–41]. To assess if the ip immunization regimen with HK GAS-M1 induces memory Th cells with such protective function, a depleting anti-CD4 monoclonal antibody (mAb) was used. One single dose of anti-CD4 mAb efficiently depleted CD4^+^ T cells, as assessed in spleen one and four days later, but did not reduce the percentage of CD8^+^ T cells (Fig. S1A and B). We administered the anti-CD4 mAb to immunized and control mice 2 days prior to lethal infection and then every third day for the whole duration of the experiment. As shown in Figure 3a, immunized CD4^+^ T cell-depleted animals were protected against the lethal dose to the same extent as non-depleted animals. This was not due to inefficient depletion of CD4^+^ T cells, as demonstrated by the absence of CD4^+^ T cells in immunized animals surviving until the endpoint of the experiment (Figure 3b). While this experimental set-up does not exclude the requirement for T cell help during immunizations, we conclude that the response that protects following administration of the lethal dose is independent of T cell help.

### Acquired protection in immunized mice is independent of adaptive immunity

**Figure 4.**
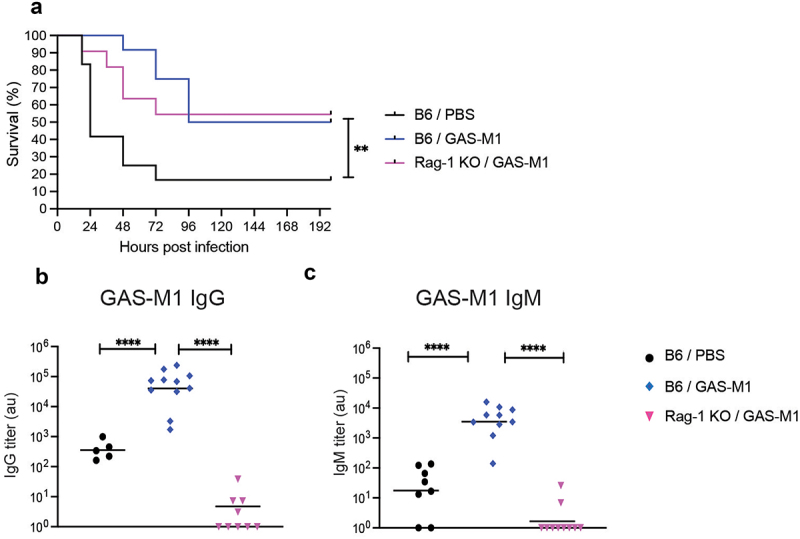
Acquired protection in immunized mice does not depend on B and T cells. B6 (*n* = 10) and Rag1^−/−^ (*n* = 10) mice were immunized ip three times with HK GAS-M1 with three weeks interval between each dose. Control B6 mice (*n* = 10) received three doses of PBS only. Three weeks after the last immunization, all mice were infected ip with 10^8^ CFU GAS-M1 and monitored for eight days. Results are pooled from two separate experiments. (a) Kaplan-Meier survival plot with log-rank tests. (b-c) Levels of serum IgG and IgM against intact GAS-M1 three weeks after the last immunization and one day prior to administration of the lethal dose. Statistical significance was analysed by one-way ANOVA with Tukey’s multiple comparisons test. *p < 0.05, **p < 0.01, ***p < 0.001 and ****p < 0.0001, ns = not significant, au = arbitrary units.

The development of B and T cells requires the expression of RAG1 and RAG2, enzymes that mediate the rearrangement of the V(D)J segments of the B (i.e. immunoglobulins) and T cell receptors [42]. In animals that lack *Rag1*, development is arrested at the pro-B/pro-T cell stage, leading to a severe deficiency in mature B and T cells [43], and an inability to generate immunoglobulins [44–46]. To assess the overall contribution of adaptive immunity to protection in the ip HK GAS-M1 immunization model, *Rag1*-deficient and control B6 mice were immunized three times with HK GAS-M1 and then injected with a lethal dose of live GAS-M1 three weeks after last immunization. Strikingly, we observed no difference in survival between B6 and *Rag1*-KO, and both groups exhibited increased protection as compared to non-immunized mice (Figure 4a). Importantly, and as similarly observed previously in BALB/c vs BALB/c-SCID mice [47], non-immunized B6 and Rag1-KO mice were equally sensitive to GAS infection (Fig. S2). As expected, we could not detect specific IgG or IgM in immunized *Rag1*-KO mice, whereas immunized B6 mice showed a considerable increase in both classes of antibodies (Figure 4b,c). Thus, our data suggest that adaptive immunity, i.e. B cells, T cells, and specific antibodies, are redundant for the protective immunity conferred by repeated ip immunization with HK GAS-M1.

### Selective increase in monocytes/macrophages and altered cytokine profile in immunized mice following lethal challenge with GAS-M1

**Figure 5.**
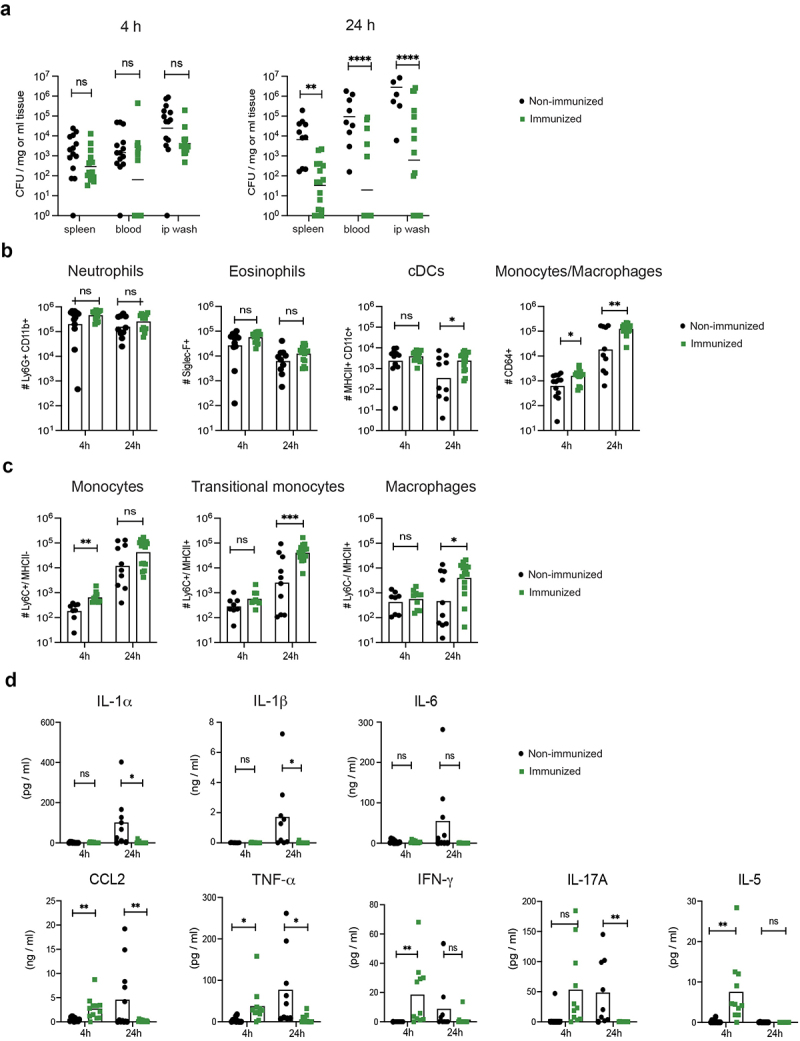
Reduced bacterial spread, elevated monocyte/macrophage numbers and an altered cytokine profile in peritoneum of immunized mice. B6 mice were immunized ip three times with HK GAS-M1 (*n* = 31) or received 3 × PBS only (*n* = 23). Three weeks after the final immunization, all mice were infected ip with 10^8^ CFU of GAS-M1. Mice were euthanized either 4 or 24 hours post-infection for analysis. (a) Bacterial loads in the spleen, blood and peritoneal cavity (ip wash). (b) Numbers of neutrophils, eosinophils, dendritic cells and CD64^+^ cells of the monocyte/macrophage lineage in the peritoneal cavity (ip wash) were determined by flow cytometry and total cell count. (c) Subset analysis of the peritoneal CD64^+^ cells by flow cytometry, showing numbers of Ly6C^+^MHCII^−^ monocytes, Ly6C^+^MHCII^+^ transitional monocytes and Ly6C^−^MHCII^+^ macrophages. (d) Concentration of CCL2 and indicated cytokines in the peritoneal cavity (ip wash). Pooled results from three separate experiments are shown. Statistical significance was analysed by unpaired t-test comparing the mean of each group. *p < 0.05, **p < 0.01, ***p < 0.001 and ****p < 0.0001, ns = not significant.

As we found that protection in HK immunized mice was independent of adaptive immunity, we next analysed the potential contribution of more immediate innate responses upon infection in immunized mice. Immunized mice were again infected with a lethal dose of GAS-M1, but now euthanized 4 or 24 hours post infection (Fig S3A) and analysed for bacterial dissemination as well as innate immune cell populations (gating strategy Fig S3B) and cytokine profiles. Bacterial loads were similar in the peritoneal cavity, blood and spleen of immunized and non-immunized mice 4 hrs post-infection (Figure 5a). However, a significant increase in colony forming units (CFUs) were detected for all organs in non-immunized relative to immunized animals after 24 hrs (Figure 5a), demonstrating that early responses in immunized mice efficiently limit bacterial propagation. Focusing on the infiltration of immune cells into the peritoneal cavity of immunized and non-immunized mice, we observed no significant differences in numbers of neutrophils or eosinophils between the groups or time points (Figure 5b). Cells belonging to the monocyte/macrophage lineage, collectively identified as CD64^+^ cells lacking expression of lineage markers for T cells (CD3), B cells (B220) and NK cells (NK1.1), were relatively few 4 hrs post-infection but showed a dramatic increase in both groups 24 hours post-infection. Notably, at this time point CD64^+^ cells were >10-fold higher in immunized vs non-immunized animals (Figure 5B and S3). In both steady-state and during inflammatory conditions, classical monocytes extravasate from the blood as Ly6C^+^ cells lacking expression of MHC class II (MHCII). They then undergo *in situ* differentiation into macrophages by a process where they first gain expression of MHCII (transitional monocytes; Ly6C^+^ MHCII^+^) and subsequently down-regulate Ly6C (macrophages; Ly6C^−^ MHCII^+^). A more detailed phenotypic analysis of the peritoneal monocytic cell population revealed that its pronounced increase in immunized animals was mostly due to an accumulation of transitional Ly6C^+^ MHCII^+^ monocytes, with a minor contribution of fully differentiated Ly6C^−^ MHCII^+^ macrophages (Figure 5C). Collectively, these results indicate that a specific increase in monocytes and monocyte-derived macrophages occur after infection and that this is significantly enhanced by the ip immunization regimen.

Cytokine production was assessed and exhibited similar profiles in the peritoneal cavity (Figure 5d) and blood (Fig. S4A) of immunized and non-immunized mice. For both groups of mice, the pro-inflammatory cytokines IL-1α, IL-1β and IL-6, which are expected to increase as a part of the early innate immune response to infection, were not detected 4 hrs post-infection, but increased in the peritoneal cavity of non-immunized mice at 24 hours. Curiously however, these cytokines were not elevated in immunized animals at this time point in spite of the increased presence of monocytes and macrophages (see Figure 5b,c) and protection against infection (see Figure 1c, 3c, and 4a). The chemokine CCL2 (MCP-1) is a key chemoattractant for monocytes [48]. In line with this function, CCL2 was selectively detected in immunized mice at 4 hrs (Figure 5d) – preceding the marked accumulation of monocytes/macrophages 24 hrs post infection in these animals (Figure 5b). In contrast, at 24 hrs post infection CCL2 was elevated in most of the non-immunized mice but could not be detected in the immunized group. A very similar pattern – i.e. an early production followed by decreasing levels in immunized mice and a late appearance in unimmunized animals – was observed for TNF-α, IFN-γ and IL-17A. The cytokine IL-5 was also selectively present in immunized mice at the 4 hrs time point but could not be detected at 24 hrs in any of the groups. Importantly, in immunized mice, all cytokine levels returned to baseline before administration of the infection dose (Fig. S4B). Collectively, these results indicate that the immunization regimen promotes an accelerated production of CCL2, TNF-α IFN-γ, IL-17A and IL-5 upon subsequent infection, while limiting the accumulation of the pro-inflammatory cytokines IL-1α, IL-1β and IL-6.

### Protection against GAS infection in immunized mice is dependent on macrophages

**Figure 6.**
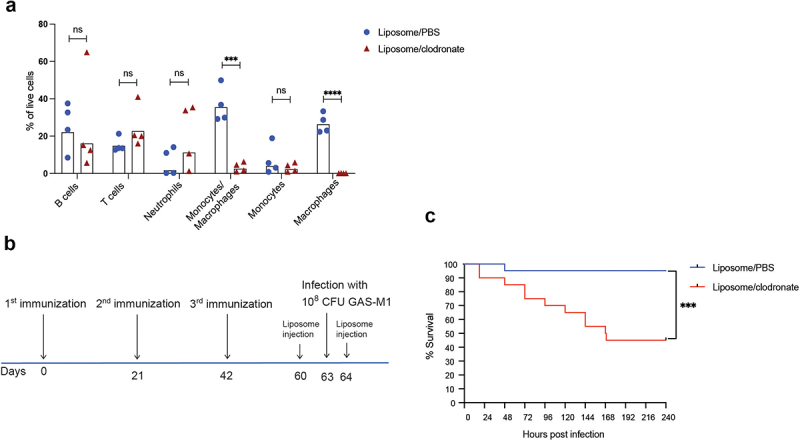
Macrophages are required for protection against GAS-M1 infection in immunized mice. (a) Assessment of macrophage-depletion by clodronate-liposomes. Mice (*n* = 4 per group) were injected ip with clodronate-liposomes (2.5 mg/mouse) or pbs-liposomes (2.5 mg/mouse) and cellular composition in the peritoneal cavity was assessed by flow cytometry 3 days later. Results show percentage of indicated subset among total viable cells and are pooled from two separate tests. Statistical significance was analysed by unpaired t-test comparing the mean of each group. (b-c) B6 mice were immunized three times with HK GAS-M1 and then challenged with lethal infection three weeks after last immunization. One group (*n* = 21) received an ip injection of clodronate-liposomes both three days before and one day after lethal infection while the control group (*n* = 21) received pbs-liposome injections at the same time points. Mice were monitored for 10 days post-infection. Results are pooled from three separate experiments. (b) Experimental layout. (c) Kaplan-Meier survival plot with log-rank tests. *p < 0.05, **p < 0.01, ***p < 0.001 and ****p < 0.0001.

Previous studies of acute infection in murine models suggest that macrophages are essential for clearing primary (non-lethal) GAS infections [49,50] As we observed a significant and selective increase in monocytes/macrophages in the peritoneal cavity after ip infection of immunized mice, we hypothesized that these cell populations may play an essential part in protection against lethal infection also in immunized animals. Macrophages can be specifically depleted using liposome-encapsulated clodronate [51]. Injected liposomes are taken up through endo- or phagocytosis, and after vacuolar fusion with lysosomes the clodronate is released and induces apoptosis of the cell [52]. In clodronate treated mice, macrophages were effectively and specifically depleted from the peritoneal cavity, as determined 3 days after liposome injection (Figure 6a and Fig. S5). We then treated immunized mice with ip injections of clodronate 3 days prior to and one day post lethal dose to ensure absence of macrophages throughout the infection, control mice received liposomes containing PBS only (Figure 6b). While control mice remained protected, clodronate treated animals exhibited significantly reduced ability to withstand infection (Figure 6c), suggesting that macrophages play an essential part in protection against GAS infection also in animals where protective immunity has been established using our ip immunization regimen.

### IFN-γ is a key determinant of protection against GAS infection in immunized mice

**Figure 7.**
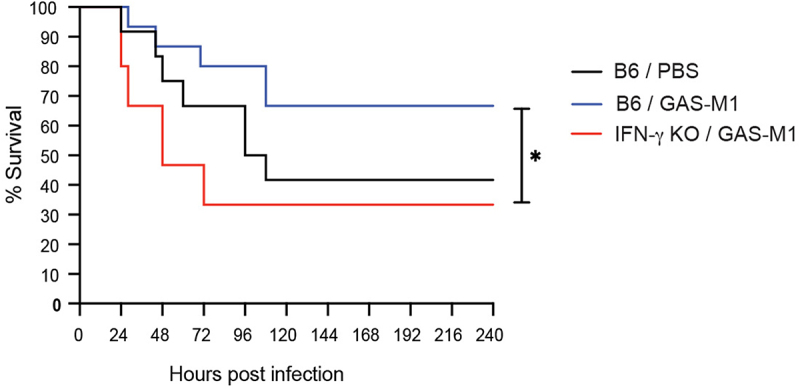
IFN-γ is required for protection against GAS-M1 infection in immunized mice. B6 (*n* = 15) and IFN-γ knock-out (*n* = 15) mice were immunized ip three times with HK GAS-M1 with three weeks between each dose. Control B6 mice received three doses of PBS only (*n* = 12). Three weeks after the last immunization, all mice were infected ip with 10^8^ CFU GAS-M1 and monitored for 10 days. Results are pooled from two separate experiments. Kaplan-Meier survival plot with log-rank tests is shown. *p < 0.05.

In immune mechanisms of protection against infection, activated Th1 cells are a major source of the cytokine IFN-γ, potentiating macrophage capacity to eliminate intracellular pathogens [53]. IFN-γ has however also been implicated in the more acute innate defences against infection with extracellular pathogens, including GAS [54,55]. Given the rapid increase in peritoneal IFN-γ levels in immunized mice following administration of the lethal dose (see Figure 5d), we assessed protection in IFN-γ deficient mice and found that in the complete absence of IFN-γ, even immunized animals succumbed to GAS-M1 infection (Figure 7). This result implies a key role for IFN-γ in infection control also in animals that have mounted protective immunity through our immunization regimen.

## Discussion

In this study, we demonstrate that recurring injections with HK wt GAS-M1 into the murine peritoneal cavity generates protective immunity. Further we show that although the ip immunization regimen induced production of GAS-specific IgG, several lines of evidence suggest that adaptive immune mechanisms such as GC development, CD4 T cells, and antibodies, are redundant for protection. In protected animals we observed an altered cytokine response upon infection, and an increased acute influx of monocytes and subsequent transitioning into macrophages. Interestingly, protection was, as previously observed in non-immune animals, dependent on the presence of macrophages during the infection phase, and surprisingly on the cytokine IFN-γ although CD4 T cells were not required (Th1 cells are commonly a major source of IFN-γ in recalled immune responses). These findings suggest that our immunization model stimulates the production of non-protective antibodies, and that the control of subsequent infection might instead be driven by memory forms in innate immune cell populations.

The generation of non-protective antibody responses is a well-known phenomenon with clear implications e.g. for approval of vaccines based on correlates of protection (instead of full efficacy trials) [56,57]. Whether or not produced antibodies confer protection may be dependent on multiple variables not only including antibody titres, but also e.g. generated antibody subclass, epitopes targeted by the response as well as the function and stability of the targeted epitope(s) [6,7,31,58–61]. Our previous work in a subcutaneous (sc) murine model showed that immunization using wt GAS induced high levels of non-protective antibodies. Immune protection required the insertion of a dominant CD4 T-cell epitope in a bacterial surface protein, specifically leading to production of antibodies of the IgG2c subclass [31]. The disparity we observed in how protective immunity is generated in our sc and ip models also further highlights previous observations suggesting that immune responses and subsequently induced protection may be radically different in distinct tissues. With special reference to GAS infections, Mortensen *et al* [29] found that adjuvanted sc GAS immunizations induced protection against a lethal ip challenge, while providing no protection in the nasal mucosa. Intranasal immunizations did however induce local mucosal protection. It has also been demonstrated that intravenously (iv) recalled Th cell responses after intranasal GAS immunizations are dominated by IL-17 producing Th [16] cells, while sc or iv immunization promote a Th1 (IFN-γ recall response [30].

The concept of memory forms in innate immune cells was first realized with early observations that live attenuated vaccines, most notably the measles vaccine and BCG, conferred heterologous protection leading to reduced incidence of several common diseases in vaccinated children [62–65]. Of particular interest for our work are a handful of studies using Gram-positive encapsulated bacteria that suggest e.g. that *Staphylococcus aureus* sc injections induce trained immunity that protect against subsequent local skin infection [66–68], and that *Streptococcus pneumoniae* intranasal immunizations elicit NK-cell mediated protection against a lethal bacterial dose [69]. Notably, recent findings in a model of mucosal vaccination using the GAS-derived peptide J8 suggested that immune protection was antibody-independent and instead relied on CD4^+^ T cells, IL-17, macrophages and neutrophils [70].

Studies of memory phenotypes in innate immune cells *in vitro* have shown that different populations present with distinct reaction phenotypes, e.g. trained monocytes and macrophages produce elevated levels of IL-6, while memory-like NK cells often present with increased production of IFN-γ [11]. In a whole organism context *in vivo* these cell-specific distinctions are not as easily discerned, and in our model the source of different cytokines can at this stage only be presumed. Indeed, we were curious to observe that protection was dependent on macrophages in immunized mice, although these animals did not present with an obvious total cytokine profile agreeing with what is expected from re-called trained macrophages. Instead, we detected accelerated and increased levels of IFN-γ (and IL-5 and IL-17), combined with an apparent absence of classical pro-inflammatory cytokines, including IL-6, upon infection of immunized mice. While the cellular source(s) of these cytokines remains elusive, the cytokine profile imprinted by the ip immunizations seems to be in line with the involvement of ILC populations, including IFN-γ producing memory-like NK cells, rather than myeloid subsets [71,72]. IFN-γ producing tissue resident NK cells are indeed present in the peritoneal cavity [73] and it seems possible that NK cell-derived IFN-γ increases the bactericidal capacity of macrophages in the immunized mice, thus restricting bacterial growth and dissemination, and promoting survival after infection [74,75]. We note that increased production of the monocyte-recruiting chemokine CCL2 [48] has previously been observed in restimulated trained innate immune cells or in peripheral tissues in *in vivo* models of trained immunity [76–79]. Our results also show elevated and accelerated CCL2 production in the immunized mice, and it seems possible that this may explain the enhanced recruitment of monocytic cells to the peritoneal cavity following the challenge with live bacteria. Finally, we are hesitant to firmly conclude that we are observing a *de facto* inhibited production of pro-inflammatory cytokines in infected immunized mice. As it is likely that the reduced bacterial proliferation in these animals result in a weaker stimulation for cytokine production, and possible that augmented consumption comes with increased levels of CD64^+^ myeloid cells, this question should be addressed in a context that allows for sufficient stratification of data.

In conclusion, our results reveal a novel aspect of how protective immunity can be generated against GAS immunization, independently of adaptive memory responses. We found that protected (immunized) animals exhibited an altered cytokine profile and increased recruitment of monocytes and their local differentiation into macrophages upon subsequent infection. Future mechanistic studies addressing memory forms in innate immune cell populations as well as the origin and function of distinct mediators in immunized mice should shed further light on the role and requirements of protective innate responses in GAS infections and vaccination strategies.

## Supplementary Material

S1_Fig.jpg

S2_Fig.jpg

S3_Fig .jpg

Author Checklist _Emami et al_rev.pdf

S4_Fig .jpg

S5_Fig.jpg

## Data Availability

The data that support the findings of this study in this manuscript and its supplemental materials are available at Open Science Framework, DOI 10.17605/OSF.IO/VQ5WR.
