# Supplementary figures and images for: Protection acquired upon intraperitoneal group a Streptococcus immunization is independent of concurrent adaptive immune responses but relies on macrophages and IFN-γ
Source: Virulence. 2025 Feb 8;16(1):2457957. doi: 10.1080/21505594.2025.2457957 (PMC11810095; doi:10.1080/21505594.2025.2457957)

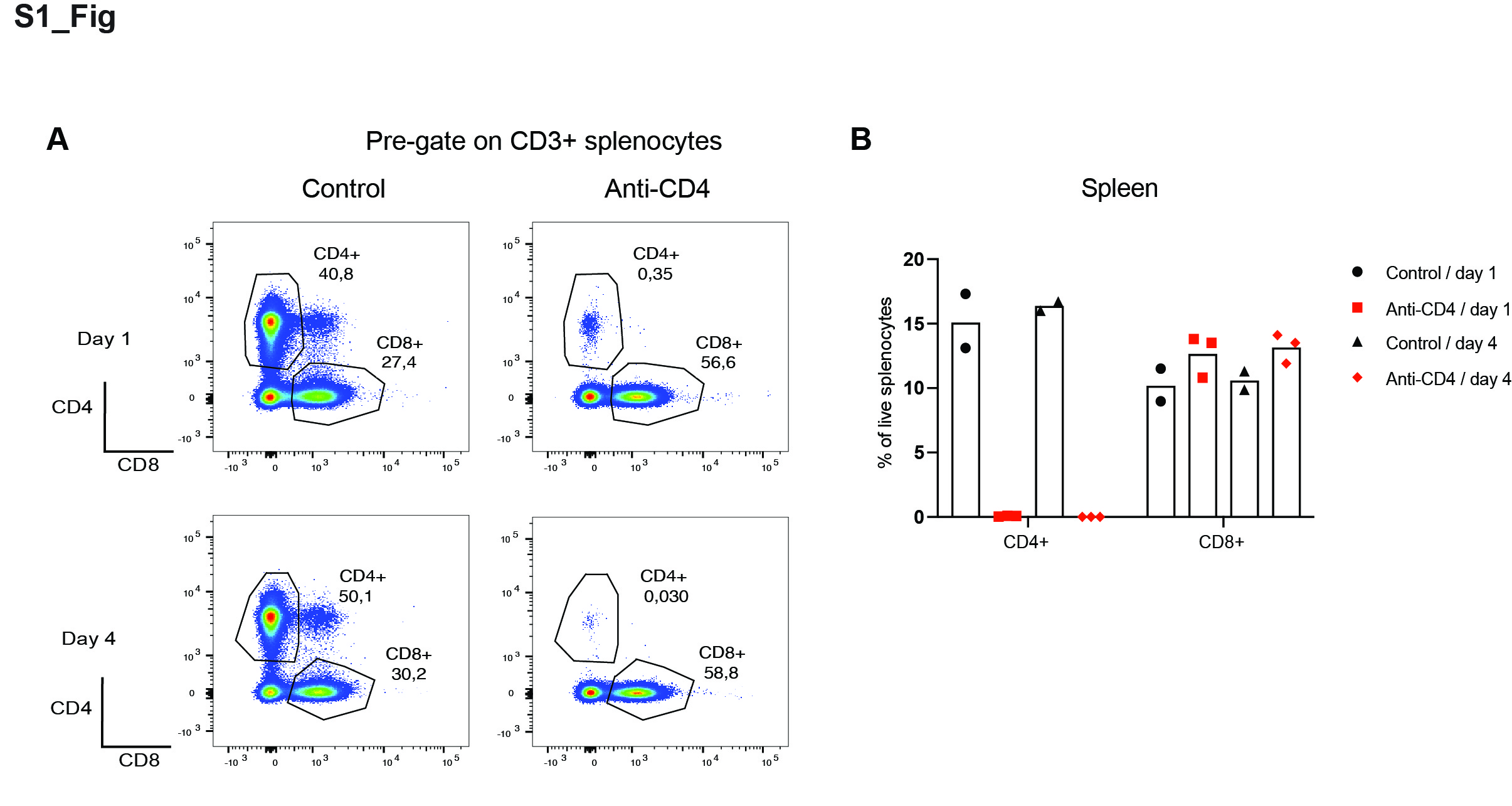

Supplement: S1_Fig.jpg [file KVIR_A_2457957_SM0053.jpg]

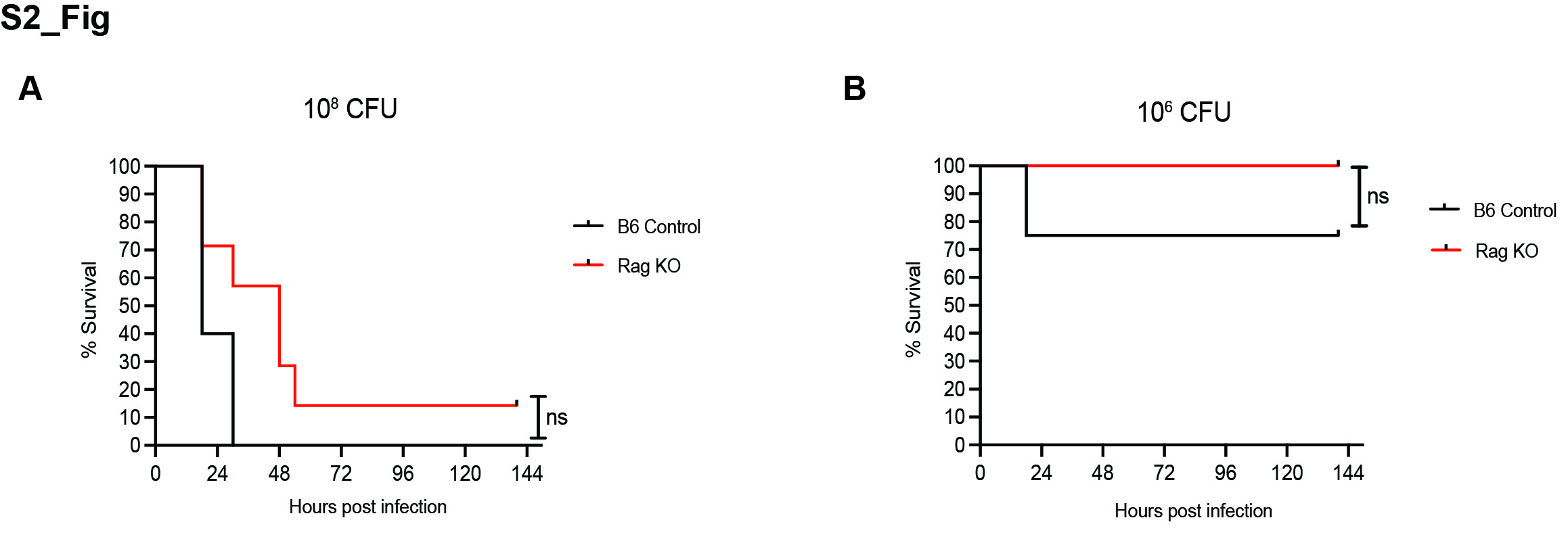

Supplement: S2_Fig.jpg [file KVIR_A_2457957_SM0052.jpg]

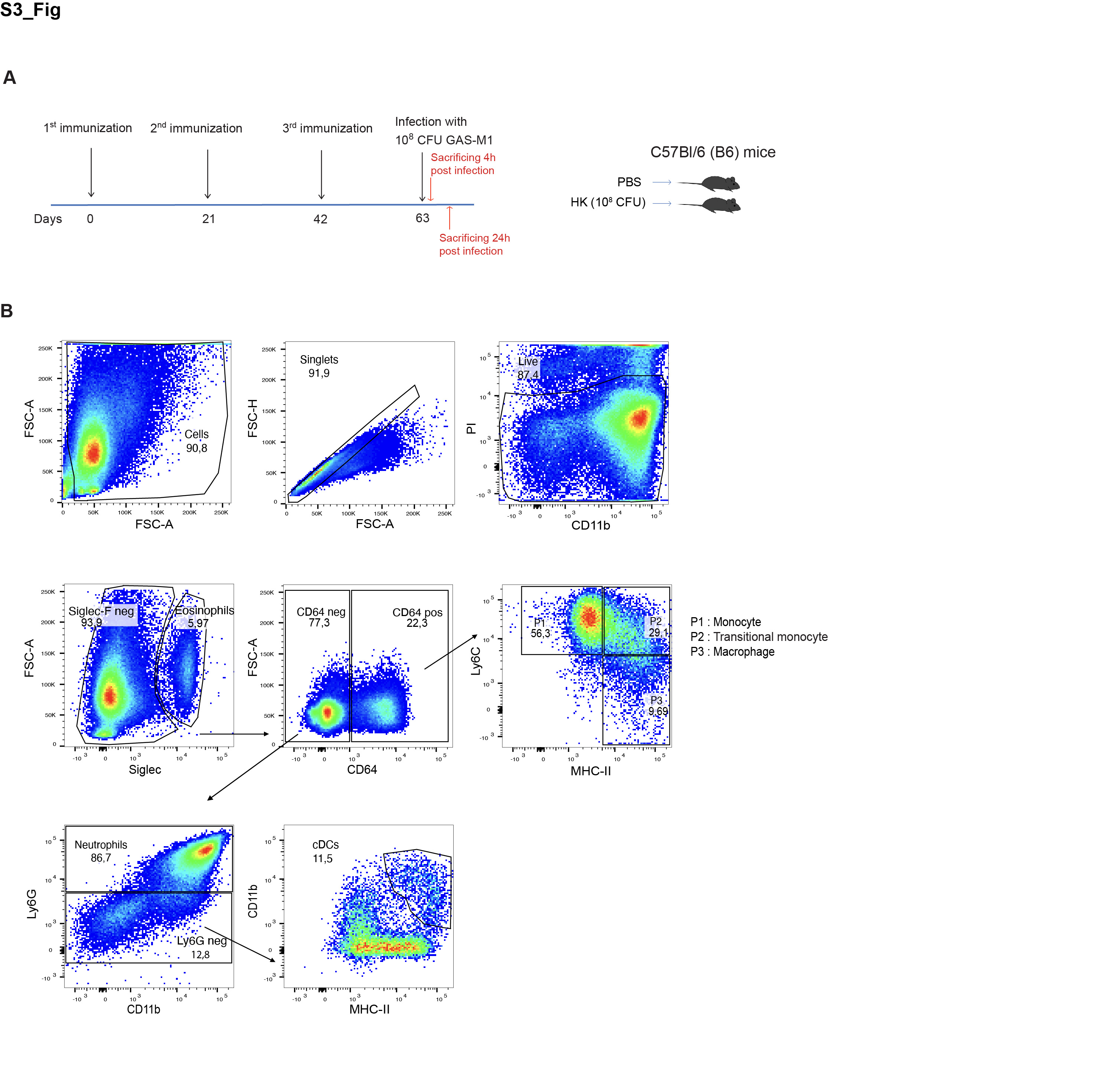

Supplement: S3_Fig .jpg [file KVIR_A_2457957_SM0051.jpg]

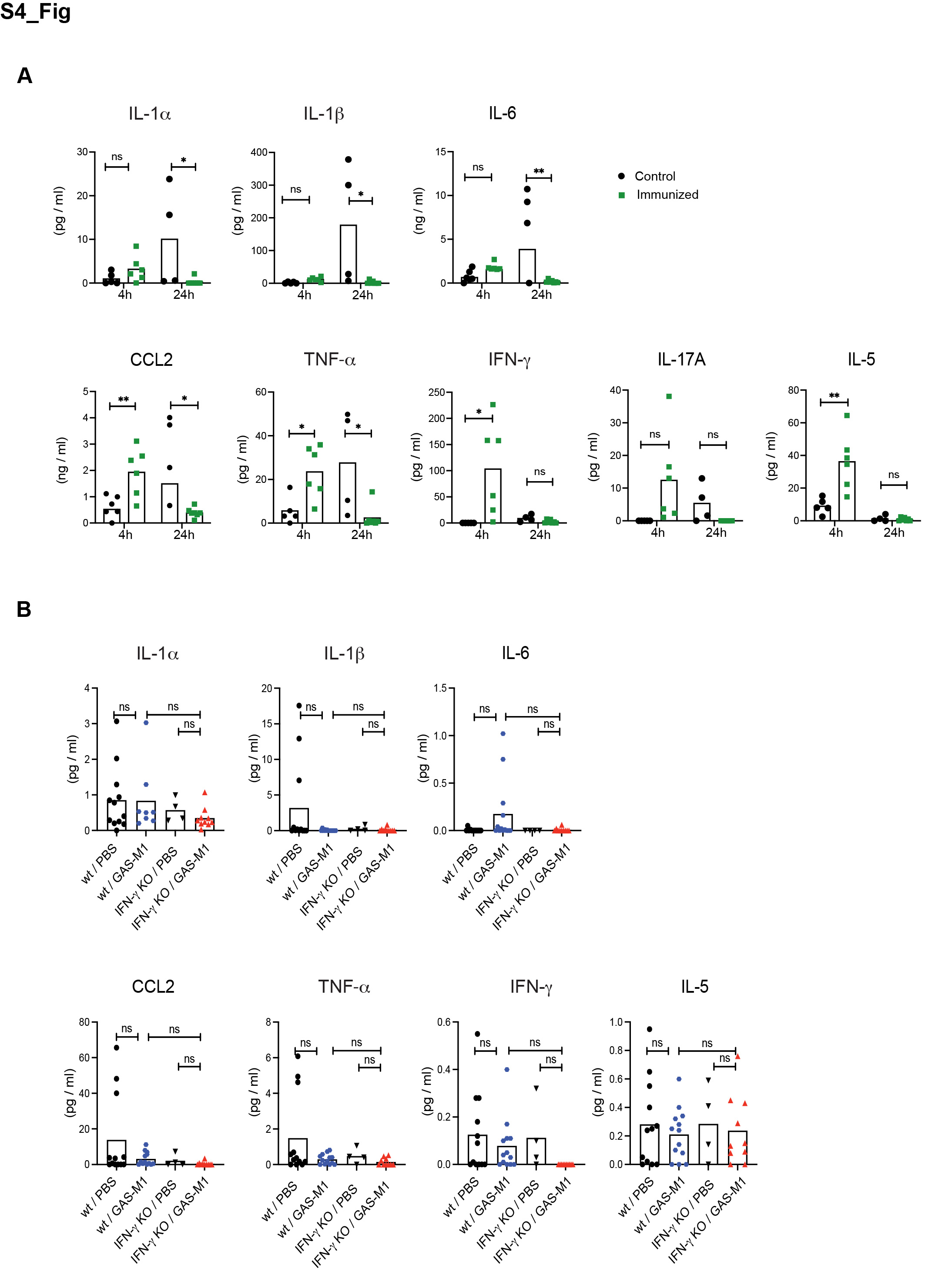

Supplement: S4_Fig .jpg [file KVIR_A_2457957_SM0049.jpg]

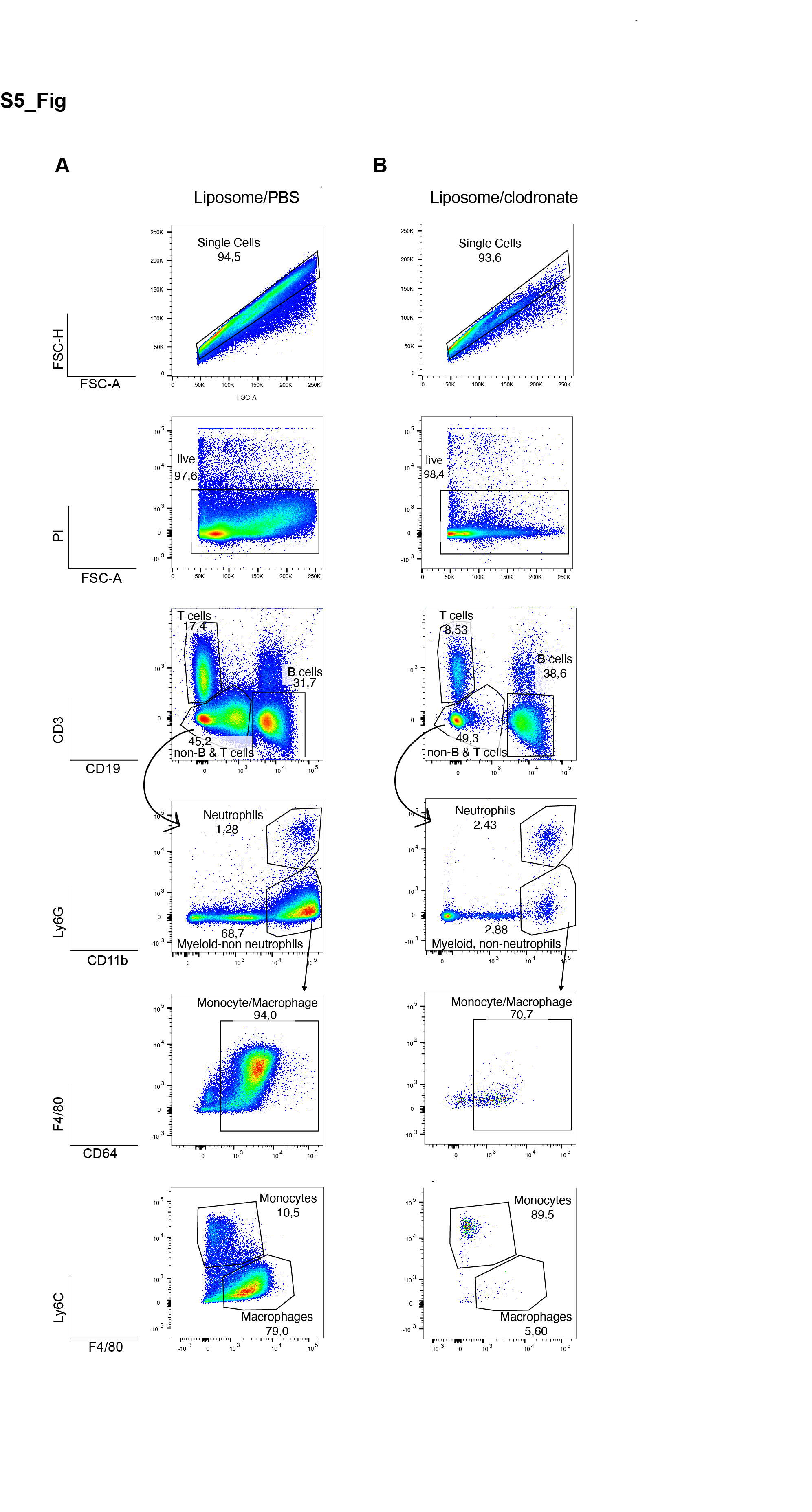

Supplement: S5_Fig.jpg [file KVIR_A_2457957_SM0048.jpg]
